# Epistemic circularity and measurement validity in quantitative psychology: insights from Fechner’s psychophysics

**DOI:** 10.3389/fpsyg.2024.1354392

**Published:** 2024-05-21

**Authors:** Michele Luchetti

**Affiliations:** Max Planck Institute for the History of Science, Berlin, Germany

**Keywords:** quantification, Fechner, psychophysics, psychology, measurement, validity

## Abstract

The validity of psychological measurement is crucially connected to a peculiar form of epistemic circularity. This circularity can be a threat when there are no independent ways to assess whether a certain procedure is actually measuring the intended target of measurement. This paper focuses on how Fechner addressed the measurement circularity that emerged in his psychophysical research. First, I show that Fechner’s approach to the problem of circular measurement involved a core idealizing assumption of a shared human physiology. Second, I assess Fechner’s approach to this issue against the backdrop of his own epistemology of measurement and the measurement context of his time. Third, I claim that, from a coherentist and historically-situated perspective, Fechner’s quantification can be regarded as a first successful step of a longer-term quantification process. To conclude, I draw from these insights some general epistemological reflections that are relevant to current quantitative psychology.

## 1 Introduction

The historical development of psychology as a science has been closely intertwined with the reflection on what counts as a psychological measurement. Several innovative developments in measurement theory over the twentieth century have directly stemmed from the work of psychologists and psychometricians, such as L. L. Thurstone, D. T. Campbell, S. S. Stevens, and R. D. Luce. Still today, the meaning and validity of psychological measurements represents a central concern for methodologists of quantitative psychology, to the point that some critics have questioned the very legitimacy of psychology as a quantitative discipline (e.g., Michell, 1997, 1999, 2008, 2012). Indeed, despite the use of quantitative methods is widely established in several areas of psychology, foundational conceptual and epistemological questions concerning the quantitative status of psychological entities and the use of quantitative methods in psychology are far from being settled.

In the period spanning from the origins of psychology as a quantitative science, in the second half of nineteenth century, up to the beginning of the twentieth century, the effort toward quantification concerned mainly two areas: psychophysics and mental testing (cf. Hornstein, 1988). Both areas were faced with the challenge of quantitatively representing characteristics, such as sensation and intelligence, which could not be directly observed. The impossibility to measure these characteristics directly,^1^ opened fundamental questions relative to what kind of measurement proxies could be considered as informative about the characteristic of interest and on what epistemological basis. In this paper, I focus on the early history of one of these enterprises, viz., Fechner’s psychophysical project of quantifying sensation. Fechner’s work can be regarded as a methodological laboratory for quantitative psychology, in that he engaged very early on with foundational measurement problems which became central to both psychophysics and psychology in general.

Fechner’s philosophy of science and his theory of measurement were quite sophisticated. Since they have been extensively analyzed elsewhere, providing an overarching account of either of the two is beyond the scope of this contribution.^2^ Instead, I will put one specific aspect of Fechner’s approach to measurement at the center of my analysis, that is, his way of addressing the problem of epistemic circularity in measurement. This is the issue of how scientists justify their belief that certain measurement procedures identify a quantity or property of interest in the absence of independent methods to assess these procedures. This issue was a central concern for the success of his psychophysical project, as it is to current discussions on the validity of psychological measurements. Therefore, examining Fechner’s work can, in my view, provide us with valuable insights to reflect on how to frame and address this problem from an epistemological perspective.

Before turning to my analysis, some important considerations are in order. Fechner’s psychophysical project aimed at providing a quantification of experience, which he operationalized as the intensity of the internal sensations produced by physical stimuli. Therefore, it may be asked to what extent we can draw a fruitful comparison between epistemological issues concerning, respectively, the measurement of sensations of physical stimuli and the measurement of more complex psychological properties, such as intelligence or memory. The possibility of such an inferential step is connected to questions concerning the nature of psychological kinds and the definition of psychological constructs. On the one hand, psychological kinds seem to be quite different from other natural or scientific kinds, in that they are very multifaceted, their causal interactions produce effects that vary highly depending on context, and they undergo constant change. Therefore, psychological constructs seem to be better characterized as concepts representing clusters, or networks, or features of phenomena, rather than as monolithic attributes (Feest, 2017, 2022a). In addition, psychological constructs should reflect the changeability of psychological phenomena and be changeable themselves (Hanfstingl, 2019). Indeed, these features represent some of the central challenges to quantification in psychology (Uher, 2020, 2021a).

Fechner’s challenge was that of finding ways to express “the amount of a psychological attribute with respect to something that was related to it in a spatio-temporal sense” (Briggs, 2021: p. 32), that is, a way to relate our internal experience, viz. sensation, to an external perceptible standard. In his view, as I will discuss, this could be tackled in the same way as for physical measurement, since he rejected any reason to restrict measurement to physical properties. However, we can see, even intuitively, that constructs like intelligence or memory are more complex and multi-dimensional than sensations. This is because these constructs refer to psychical performances which emerge through the joint manifestation of several different abilities, such as verbal knowledge, reading comprehension, etc (Toomela, 2008). Most importantly, the methods by which we can access these different phenomena vary, depending on the nature of the phenomena themselves. The response to physical stimuli can be studied through *extraquestive* methods, based on the possibility of establishing a shared perception of a physical phenomenon, both internal and external to individuals’ bodies (Uher, 2019). However, these methods are not available for the study of internal psychic phenomena, that can be perceived only by each individual. These must be studied through *intraquestive* methods, which necessarily rely on language and interpretation by both the individuals acting as measurement instruments (the raters) and the scientists.^3^

In sum, features related to the multi-dimensionality and complexity of the psychological subject matter worsen the impact of certain general issues, such as those related to the possibility of experimental control (Trendler, 2009; Wajnerman-Paz and Rojas-Líbano, 2022).^4^ On the other hand, psychological measurement presents specific conceptual, methodological, and epistemological challenges, compared to sensory measurement, due to both the peculiar nature of the phenomena under investigation and the limitations characterizing the appropriate methods currently available to study them.^5^ Nonetheless, this does not mean that some fundamental issues characterize both sensory measurement and the measurement of more complex psychological phenomena. Indeed, the problem of epistemic circularity in measurement represents an issue that, despite manifesting itself in different ways and with different intensities, concerned both Fechner’s sensory measurement and contemporary quantitative psychology. Given this level of abstraction, my insights on Fechner’s approach to this problem will not translate into methodological maxims directly applicable to current psychological measurement. Rather, it will provide some broad epistemological considerations relative to two specific aspects: (1) the role of implicit untested measurement assumptions; (2) what counts as successful measurement and how it impacts general epistemic categories like validity and objectivity.

In section “2 Epistemic circularity and psychological measurement,” I will introduce the epistemic problem of circular measurement, focusing specifically on psychological measurement and its challenges. In section “3 Fechner’s psychophysics and the making of sensation as a quantity,” I will first present Fechner’s psychophysical research program in general and then zoom in on his approach to the problem of circular measurement. In section “4 Epistemological insights from Fechner’s quantification of sensation,” I will develop the main argument. First, I will focus on some relevant objections to Fechner’s quantification of sensation raised by both his contemporaries and more recent commentators. Then, I will analyze Fechner’s approach to measurement circularity and I will discuss it against the backdrop of Fechner’s broader epistemology of measurement. Finally, I will reconsider Fechner’s contribution vis-à-vis the subsequent history of psychophysical measurement. Section “5 The relevance of Fechner to current methodology of psychological measurement” will conclude by offering some insights on how the present work is relevant to contemporary quantitative psychology.

## 2 Epistemic circularity and psychological measurement

From an epistemological point of view, the problem of what counts as a good, reliable, or accurate measurement is connected with the problem of how to appropriately identify the target of measurement, that is, which concepts or constructs appropriately represent the measurand (Tal, 2019). These issues have indeed been a central focus of methodological debates in psychological measurement. However, I will first present how they have been tackled in recent philosophical and metrological literature as issues that concern measurement across the sciences.

Measurement procedures are often described as concrete interactions between one or more epistemic subjects (observers and/or test subjects), a material apparatus, and some phenomenon occurring in an environment. Examples of this are when we observe the mercury dilate in the column of a thermometer hanging on the wall or when a person responds to a standardized item on a personality test questionnaire. In the first case, the physical process itself that takes place during the measurement interaction can also be used to *represent* a certain relationship between quantities, as when we read a measurement of temperature out of an indication of the length reached by the mercury in the thermometer column. In the second case, the measurement interaction presupposes a certain representational relationship between measured items and certain target properties, as when scores attributed to individual responses of a personality test questionnaire are taken to be informative about a certain personality trait.

**FIGURE 1:**
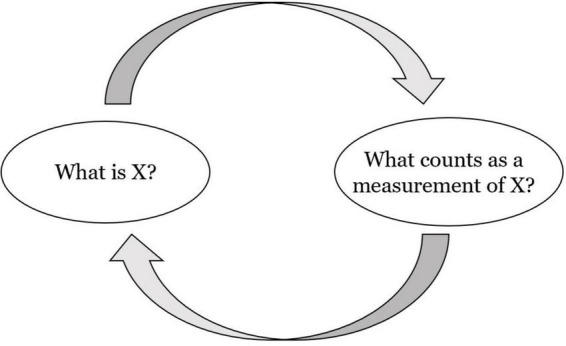
A graphic representation of the general problem of epistemic circularity in measurement. The arrows represent the direction of epistemic access.

The fact that measurement has both a material and a representational dimension is central to an epistemic conundrum, namely, the problem of circular measurement.^6^ This is the issue of how scientists justify their belief that certain measurement procedures identify the quantity or characteristic of interest in the absence of independent methods to assess these procedures. In the case of measuring a physical quantity, for example, we often infer its value from the values of other quantities, as when we infer measurement outcomes of temperature from indications of length of a thermometer column. This inference is based on knowledge of the empirical relationship between the quantities of temperature and length in a specific physical interaction. However, knowledge of this relationship is itself a scientific achievement, which may seem impossible to attain without the use of evidence previously acquired through measurements. Hence, the risk of circularity (Figure 1), since answers to the questions “What counts as a measurement of X?” and “What is X?” often seem to presuppose one another when a theoretical understanding of the quantity or characteristic of interest is weak.^7^ This means that the risk is more likely to occur when knowledge of the empirical relationship among the representing quantity and the represented quantity is yet in the making (van Fraassen, 2008).

Recent approaches in the epistemology of measurement have suggested that the circularity itself is not vicious, if we take a historical and coherentist approach (Chang, 2004; van Fraassen, 2008, cf. Tal, 2020). Rather than trying to avoid the risk of circularity, this should be embraced as a constitutive part of the process that leads to progress in measurement. According to these perspectives, the meanings of quantity concepts emerge from a historical and iterative process of mutual feedback between theoretical advances and improvements in measurement standards. With each iteration, the quantity concept is re-coordinated to a more stable set of standards, which allows for theoretical predictions to be tested more precisely. This, in turn, enables subsequent development of theory and the construction of more stable standards, and so on. Indeed, we can only realize how this process avoids vicious circularity when we look at it either “from above,” i.e., in retrospect given our current scientific knowledge, or “from within,” by looking at historical developments in their original context (van Fraassen, 2008: p. 122).

These recent coherentist approaches to measurement have developed from a primary focus on examples from physics, hand in hand with developments in metrological discussions also primarily targeting physical measurement and engineering (e.g., Mari, 2003; Frigerio et al., 2010; Giordani and Mari, 2012). One crucial feature of these approaches is that they shift from an exclusive focus on mathematical representational structures and the definition of quantity terms typical of classic mathematical theories of measurement, like the Representational Theory of Measurement. Instead, these approaches pay substantial attention to *realizations* (cf. Tal, 2020), that is, the physical instruments or procedures that approximately satisfy certain definitions of quantities (cf. JCGM, 2012: 5.1). These coherentist perspectives have been applied to analyze how measurement circularity can emerge and be tackled even beyond the physical sciences.^8^

Metrologists and psychometricians that are in dialog with these coherentist approaches have attempted to bridge physical and psychological measurement under overarching models of measurement (e.g., Mari et al., 2016, 2023). However, the very concept of a realization as provided by the JCGM, when translated into the context of psychological measurement, implies specific and difficult challenges that have received limited consideration by the philosophical and metrological literatures just mentioned. Two of these challenges are particularly relevant to the problem of circular measurement. The first concerns the fact that identifying empirical regularities which describe the relationship between two quantities or properties in a specific measurement interaction constitutes an intrinsic challenge for psychology.^9^ The possibility to represent a characteristic that is not directly observable in terms of another observable property or quantity requires, in fact, an unbroken chain of interactions that goes from the first observable property to the measurand (JCGM, 2012). This chain of interactions is established through the identification of causal quantitative relations from the first property to the measurand. Most natural sciences can rely on shared perception as a criterion for metrological traceability, i.e., on the fact that inter-subjective agreement on what is being observed can be achieved, thus grounding the possibility to further infer causal empirical relationships among quantities. As the problem of measurement circularity shows, identifying these empirical regularities, also known as *measurement laws*, can be difficult in all sciences. While, as I will discuss, Fechner developed his quantification of sensation by adopting a standard in a spatio-temporal sense, this does not seem a viable possibility for a great part of psychology. This is mainly because its intraquestive measurement methods based on subject reports cannot support shared perception as a criterion for metrological traceability (Uher, 2019, 2020).

The second challenge concerns the fact that, in the psychological literature, realizations are often taken to refer to the questionnaires or other standardized assessment tools through which psychological measurement is performed. Therefore, according to this interpretation, it is the representational relationships among these measurement instruments, the target characteristics that they are supposed to be informative about, and the constructs that provide definitions of those characteristics, that are relevant to successful measurement. Indeed, this understanding has been for a long time at the center of discussions concerning validity, a key methodological notion for evaluating the quality of measurement and assessment tools in psychometrics.^10^ The aspect of validity that, from the 1950s, started to be called *construct validity* involves building and testing theories about psychological characteristics which we also try to empirically access via measurement (Cronbach and Meehl, 1955; Messick, 1989).^11^ One of the aims of construct validation is to clarify the definition of characteristics that are also measurement targets, so that the outcome of a certain measurement procedure can justifiably be claimed to be informative about the intended measurand, rather than about something else. Indeed, approaches based on construct validity resonate, to some extent, with the coherentist perspectives on measurement previously discussed, based as they are on a process of mutual refinement between measurement standards and theoretical concepts over time.

Yet, as both philosophers and methodologists have pointed out, conceptualizations of the relationship between theoretical constructs, the psychological phenomena that they describe, and the measurement outcomes that are supposed to be informative about them, remain underdeveloped in construct validity theories, thus leaving room for different interpretations of the meaning of test results.^12^ In addition, the tendency to focus on questionnaires and standardized assessments as the only measurement instruments can lead to underappreciate the complex epistemic role of test subjects in the measurement interaction. Indeed, psychological measurement presents us with the peculiar issue of conceptualizing humans as both objects of measurement and measurement tools, thus challenging any approach to measurement which tries to dispense from a subjective evaluative component. Fechner was a forerunner of this realization, in a trajectory that—passing through Stevens’ (1956) method of magnitude estimation based on the conception of the person as a measuring system—arrives at recent systematic perspectives on the “human as a measurement instrument” (e.g., Berglund et al., 2012; Pendrill and Petersson, 2016; Pendrill, 2019).^13^

A focus on the subjective component of measurement will be central to my analysis of Fechner’s quantification of sensation and his approach to measurement circularity. Indeed, the recent coherentist epistemologies of measurement have reminded us that a human component is present in all measurement. This is because, at some point in all histories of quantification, inter-subjective evaluation, rather than reliance on well-established quantitative relations, was the basis for accepting certain measurement standards as valid. Therefore, such a consideration is most relevant in cases where the issue of measurement circularity is a challenge to the coherence of the assumptions on which quantification is based. By relying on a coherentist perspective of measurement, I will emphasize the “human” component of Fechner’s approach to the quantification of sensation, which required him to put the subjective at the center of his quantification both methodologically and epistemologically.

## 3 Fechner’s psychophysics and the making of sensation as a quantity

Initially trained as a medical doctor, Fechner [1801–1887] became a central figure in nineteenth-century German science and culture, contributing to several fields from physics to psychology, from statistics to esthetics, from metaphysics and the theory of mind to satirical literature (Fancher, 1996; Arendt, 1999; Heidelberger, 2004). Some narratives (e.g., Boring, 1961), characterize Fechner’s psychophysics as an attempt to scientifically substantiate his philosophical view of the relationship between mind and matter, according to which the physical and the mental are two manifestations of one and the same reality (cf. Fechner, 1851/1957). Instead, several historians have emphasized the coherence of Fechner’s psychophysical research program with his broader view of scientific inquiry (e.g., Marshall, 1982; Heidelberger, 2004). In addition, they have connected Fechner’s emerging interest in psychophysics with central biographical events, such as his experience of prolonged visual deficiency and temporary mental impairment (e.g., Nicolas, 2002; Meischner-Metge, 2010).

Experiments on sensory modality had been performed from the seventeenth century, and psychophysical methods were systematically used in the work on touch carried out by Ernst Heinrich Weber [1795–1878]. Weber (1834, 1846) used comparisons between stimuli to identify thresholds of experience, that is, to identify the minimum stimulus required to perceive a sensation.^14^ Among his results, Weber showed that the stronger a stimulus, the more intense should another stimulus be so that the difference with the former can be sensed. In other words, the minimal change in stimulus required for a difference in sensation to be perceived is a constant fraction of the values of the stimulus in the background. Therefore, the smallest discernable distinction between two stimuli can be expressed as an invariable ratio between them, independently of their strength. The formula expressing this ratio is: ΔR/R = *c*, where ΔR is the relative threshold for the stimulus, that is, the limit at which the difference is discernible, R is the stimulus and *c* a constant specific to each sensory modality.

Fechner invented the term *psychophysics* to refer to the scientific study of the functional relationship between body and mind, which he had intended to pursue as an exact science well before getting acquainted with Weber’s empirical results (Marshall, 1982). Fechner conceived psychophysical processes as those physiological bodily processes immediately accompanying psychical events. Central to his psychophysical theory was the distinction between inner and outer psychophysics. Inner psychophysics focuses on the relation of the mental to the internal functions with which psychical activity is closely related, that is, on the relationship between the mental and neurophysiological activity. *Psychophysical excitation* was Fechner’s term to describe the process, occurring in the brain and in the rest of the nervous system, through which the crossing of nerve tracts generated psychical activity. Outer psychophysics, instead, focuses on the relation of the mental to the body’s external aspects, i.e., to the physiology of the senses.

**FIGURE 2:**
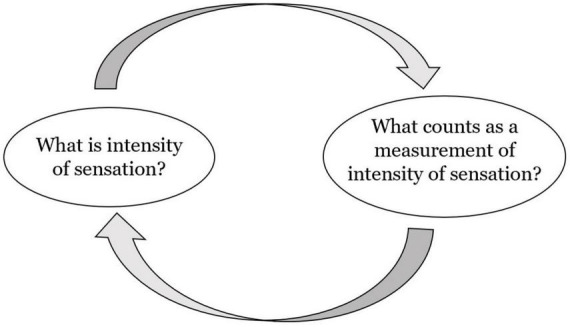
The epistemic circularity faced by Fechner in his attempt to quantify the intensity of sensation. In this case, the question “What is intensity of sensation?” includes a number of other sub-questions, including “What kind of property is intensity of sensation?” and “Is it quantifiable?”

Initially, Fechner searched for knowledge of the nervous system that would allow him to pursue inner psychophysics and, thus, directly investigate the causal processes giving rise to experience. However, he could not find such knowledge. The biophysicists working on the physical-chemical explanation of biological processes at the time were scarcely interested in the brain and the nervous system, plausibly because they did not view consciousness and higher mental activity as explainable in materialistic terms (Culotta, 1974). Therefore, Fechner’s only viable empirical access to psychophysical processes was through the use of the indirect measurement methods offered by Weber’s outer psychophysics, that is, the study of the relationship between physical stimuli and sensations. In this sense, Fechner conceived of psychophysical processes as abstract theoretical constructs when he wrote that “The mental intensity of an element is a mathematical fiction which has no other meaning than to provide for a calculation of a relationship which occurs in a system of elements” (Fechner, 1851/1957: p. 374). Yet, for this mathematical fiction to have concrete meaning Fechner had to establish a mapping between the characteristic of interest, viz., the intensity of sensation, and some measurable proxy. This mapping would ensure that his measurement methods would actually measure what he intended to measure in the absence of independent standards. Put it in another way, Fechner had to deal with the problem of epistemic circularity in measurement (Figure 2): How could he identify the “right” way of measuring intensity of sensation without already presupposing some quantitative understanding of intensity of sensation?

As I have mentioned, Weber had already established that some form of reliable measurement could be achieved in the experimental study of sensory thresholds for the different sensory modalities, by relying on the linear function relating physical stimuli and sensory thresholds that he identified. Indeed, his approach rested on identifying relative thresholds of experience based on increments of the stimulus, that is, on *ordering* sensations of different intensities according to the intensity of the stimulus that produced them. Fechner’s goal was more ambitious, in that it aimed at *quantifying* sensations, based on his firm conviction that psychical phenomena have a quantitative dimension (Fechner, 1858). To this purpose, he set out to construct a mapping between the intensity of sensory stimuli, his only available physical proxy, and his attribute of interest, viz., experience, operationalized as intensity of sensation. This mapping required establishing (i) a measurement unit that could ground a scale of intensity of sensation, (ii) a functional relationship that would justify the representation of intensity of sensation in terms of intensity of the stimulus, and (iii) a material measurement standard that would embody this functional relationship and, thus, enable the actual quantitative study of experience.

Indeed, the only material measurement standard for which a functional relationship between stimulus intensity and sensation intensity could be identified, and that could then be used to measure sensation, is the human body. The very possibility of psychophysics as a quantitative discipline in Fechner’s sense was based on the assumption of a shared human physiology, which ensures the stability of the functional dependence of sensory reaction from stimulus intensity. As I will show more in detail in section “4.2 Stabilizing the problem of measurement circularity,” this assumption was a central component of Fechner’s approach to measurement circularity.

Fechner’s first important conceptual innovation concerns how he developed a unit of measurement by using the fact, experimentally established by Weber, that the smallest discernable distinction between two stimuli can be expressed as an invariable ratio between those stimuli (ΔR/R = *c*).^15^ More precisely, Fechner used this regularity to define a *just noticeable difference* (jnd), that is, the smallest difference in sensation that corresponds to the smallest perceptible change in stimulus. In such a way, the change in stimulus used to produce a difference in sensation can be taken as a standard, i.e., a physical proxy, to measure equal units of sensation intensity. In other words, this provides a definition of the unit of a scale of intensity of sensation, which Fechner calls the *Fundamentalformel*, or basic formula: ΔE = ΔR/R *c*, where ΔE is a just noticeable difference in sensation, while the equation expresses which intensity of stimulation corresponds to a unit of sensation. To construct a measurement scale out of this definition of a psychological unit, Fechner had to make two assumptions. The first is that all jnds are of equal magnitude, that is, that they produce the same change in sensation, independently of the base value of the stimulus. The second is that the jnds can be summated in the same way as material units. Both assumptions were later to be subject to strong criticism.

Yet, the basic formula is not by itself sufficient to ground a measurement scale of sensation intensity. To that purpose, Fechner needed to identify a functional relationship that, by specifying the number of jnds that make up *all* differences in sensation, would justify the representation of intensity of sensation in terms of intensity of the stimulus. To precisely characterize this relationship, which he later called the *Maßformel*, or measurement formula (also known as “Fechner’s law”), and deploy it as a constructive principle for his scale of sensation, Fechner had to tackle the circularity problem. In other words, he had to somewhat justify that this measurement scale based on his chosen unit was actually measuring what it was supposed to measure. In the absence of independent support for his definition of the unit of sensation intensity, he set out to construct his measurement scale through a sort of bootstrapping process (Heidelberger, 2004). Having his basic formula, i.e., his definition of a unit of sensation intensity, in the background, Fechner first tested empirically the equality of sensation intensities through the method of adjustments, an experimental technique in which the test subject can adjust the intensity of the stimulus until it reaches a threshold and a just noticeable difference is perceived. Then, he statistically reduced individual aberrations in the evaluation of equality of differences (i.e., in the identification of thresholds).

The next step was to test sensations of different strength to identify which increase in stimulus is required to obtain an increase in sensation that is subjectively experienced to be identical to the others. The datapoints obtained in this phase were meant to enable Fechner to empirically validate his scale. To obtain these datapoints, Fechner adopted the method of right and wrong cases, based on comparing the weight of two containers and discriminating between the two respective physical stimuli.^16^ He only used himself as an experimental subject, but he corrected for the possibility of differences in subjective evaluation of the stimuli intensities. He did so by repeating the same comparisons several times, and then using a normal distribution to represent the probability of discriminating the stimuli. On top of being a great innovation at the time, this methodological point will be relevant to my discussion of Fechner’s approach to measurement circularity in Section “4.2 Stabilizing the problem of measurement circularity.”

Finally, Fechner expressed these datapoints as a monotone function between the increment of sensation found to be constant and the increment of stimulus required for it. In other words, Fechner moved from differences in sensation to *differentials*, i.e., infinitesimally small units of sensation. This move was necessary to express his measurement formula in logarithmic terms and use it to justify the measurement scale he constructed.^17^ The resulting measurement formula, E = *z* ⋅ logR, expresses the functional relationship between values of the representing quantity, intensity of stimulus, and the represented quantity, intensity of sensation, thus justifying the use of intensity of stimulus as a proxy for measuring intensity of sensation.

## 4 Epistemological insights from Fechner’s quantification of sensation

### 4.1 Objections to Fechner’s quantification and developments after Fechner

Fechner’s critics found several assumptions underlying his proposed quantification of sensation intensity to be highly problematic.^18^ Most critics rejected the significance of the measurement formula for inner psychophysics and focused on its role for outer psychophysics. This was not taken lightly by Fechner, who wanted his measurement formula to be regarded as an empirical law of inner psychophysics (Marshall, 1982). According to Fechner, in fact, the measurement formula has a double character. On the one hand, his functional relationship between the intensity of the stimulus and the actual target of measurement, i.e., the intensity of sensation, is based on a unit of measurement that, even though resting on Weber’s empirical regularity, *stipulates* the standard for measuring sensation. On the other, the measurement formula expressed, according to Fechner, the relation between psychophysical excitation, i.e., the physiological phenomenon causing sensation, and intensity of sensation. This part of the law remained theoretical, given that psychophysical excitation could not be empirically accessed. In addition, the assumption that led Fechner from Weber’s law to his basic formula, that is, that all jnds can be considered as equal, was particularly contested already by Fechner’s contemporaries, together with the assumption that units of sensation can be added to one another just in the same way as physical units (e.g., Tannery, 1875a,b; von Kries, 1882; James, 1890). These two criticisms, which came to be discussed together by the label of “quantity objection” (cf. Boring, 1950; Michell, 1999, 2012), emphasized the lack of independent empirical justification for the two assumptions just mentioned.^19^

Fechner’s derivation of his measurement law and his empirical method of constructing a scale by concatenating experimentally estimated units (the jnds) eventually produced a schism between physicists and psychologists in the 1930s. Their divergent assessment of whether it is possible to make a quantitative estimate of sensory events in the absence of independent measures of sensation intensity eventually led to separate paths in the development and assessment of conceptualizations of measurement throughout the twentieth century (Berglund et al., 2013). While this separation was something that Fechner himself had attempted to break, developments within twentieth-century psychophysics showed the empirical limitations of Fechner’s measurement standard. Crucially, Stevens (1956, 1957) established that Fechner’s units of sensation, the jnds, cannot be considered to be uniformly equal, as Fechner postulated. Stevens adopted the method of fractionation, a method by which the subject judges whether one weight is half that of another, or one sound twice as loud as another, etc. By making comparisons between incremental assessments of jnds and sensory experiences through fractionation, he showed that the jnds are, in fact, not uniformly equal. Fechner’s logarithmic formula was eventually replaced by Stevens’ power law, resulting from a modification of the basic formula (Stevens, 1969, 1970).^20^ While the compatibility of Fechner’s logarithmic formula with Stevens’ power law and further formulations has been, and still is, a topic of debate in psychophysics (e.g., Wasserman et al., 1979; Laming, 1991, 2010), it became clear that Fechner’s measurement formula is only applicable to a restricted range of sensory modalities. Even though Fechner’s methods have never really been abandoned (e.g., Luce and Edwards, 1958; Eisler, 1963; Falmagne, 1971; Murray, 1993), later developments downsized the validity of Fechner’s measurement standard and questioned the view of psychophysics as an enterprise aimed at discovering fundamental quantities (cf. Luce, 1972).

In the rest of this section, I will provide an assessment of Fechner’s approach to measurement circularity by situating it in a historical perspective and in relation to his conceptualization of measurement. This will enable me to provide an assessment of his psychophysical project by looking at it both “from above,” i.e., in retrospect given our current knowledge, and “from within,” by considering historical developments in their original context.

### 4.2 Stabilizing the problem of measurement circularity

In section “3 Fechner’s psychophysics and the making of sensation as a quantity,” we have seen that Fechner’s quantification of sensation intensity required presupposing a host of assumptions that were, at least at the time, untested or untestable. These included the assumptions concerning the equality and additivity of jnds, that became the focus of heated debates and are still relevant to methodological discussions today. However, much less attention has been paid to another of Fechner’s assumptions, which had a crucial role both in his experimental practice and in his approach to the problem of measurement circularity. This is the assumption that all human individuals share a common physiology.

Fechner’s approach to quantifying sensation involved using Weber’s experimental methods of outer psychophysics, which relate behavioral response data to physical stimuli, in order to gain access to inner psychophysical processes, i.e., the neurophysiological goings-on of sensory experience. The possibility of this methodological jump was justified by Fechner’s assumption of a shared human physiology. For the purposes of establishing the correlation between the mental and the physical, in fact, Fechner considered that the individual differences in the physiological make-up of test subjects were irrelevant. In addition, this assumption justified the possibility to use himself as one of few, or even the only, test subjects in his experimental practice. Epistemologically speaking, this idealizing assumption replaced the process of standardizing his measurement instrument, i.e., the human sensory apparatus.

More generally, the assumption of a shared human physiology ensured the stability of the empirical regularity black-boxed by his measurement formula, i.e., the causal relationship between the intensity of a sensory reaction and the psychophysical excitation produced by a stimulus of a certain intensity. Fechner was aware that subjective evaluation has an impact in the identification of thresholds of experience, in that it provides an important source of variability. For this reason, he characterized the notion of threshold in statistical terms.^21^ As I previously mentioned, Fechner replicated the experiments through which he established the empirical datapoints validating his measurement formula. This methodological step allowed him to control for differences in subjective judgment of the stimuli. Yet, as he was using only himself as a test subject, this step could not control for possible differences in physiological make-up. The assumption of a shared human physiology *de facto* enabled Fechner to discount the possibility of experimental variation resulting from differences in psychophysical excitation due to different neurophysiological make-ups of test subjects, the impact of which, as we have seen, would anyway be out of reach given the state of neurophysiological knowledge at his time. By anchoring the reaction to sensory stimuli to a univocal and stable causal basis, i.e., our shared sensory apparatus, Fechner could then set out to develop a representational mapping between the empirically accessible side of the functional relationship that he aimed to establish, i.e., the intensity of the stimulus, and the characteristic that was his actual measurement target, i.e., the intensity of sensation.

In this sense, the assumption of a common human physiology has a special epistemic status, since it provided Fechner with an anchor to keep the circularity problem stable. Without this assumption, the variability due to individual differences in physiological make-up would have made it much more difficult, if not impossible, to establish the functional dependence between intensity of stimulus and intensity of sensation. This is because, if that were the case, differences in reactions to the same sensory stimulus would have been considered as partly dependent on physiological differences among subjects. Yet, there would have hardly been a way to factor the extent of the causal influence due to these differences, given the insufficient neurophysiological knowledge of the time.

### 4.3 Reassessing Fechner’s standard in light of his epistemology of measurement

In addition to the assumption of a shared human physiology, the very idea that sensation itself is something that can be at all quantified was another crucial untested assumption behind Fechner’s approach to measurement circularity. Fechner’s conventional assumption of the equality and additivity of jnds has been directly invoked as the remote cause of the overly liberalized current view of quantification in psychometrics (Michell, 2006, 2008). From this perspective, Fechner stipulated his measurement standard without securing a logically prior step. That is, he did not verify empirically the quantitative character of the relationship between the characteristic of interest, i.e., the intensity of sensation, and the chosen standard, i.e., the intensity of stimulus (Michell, 2006, 2012). While engaging with this argument is beyond the scope of this contribution, in my view we can understand Fechner’s assumption of the quantifiability of sensation only against the backdrop of his nuanced epistemological perspective and from within the historical context of his measurement practice.

Some commentators have emphasized how Fechner’s approach to quantifying sensation was entangled with his correlative interpretation of measurement (Murray, 1993; Heidelberger, 2004; Briggs, 2021). According to Fechner, in fact, the relationship between the external stimulus and sensation is not a causal one. While the stimulus causes psychophysical excitation in the brain or in the nervous system, it is not directly causally related to sensation. Rather, the stimulus is only functionally linked to sensation, inasmuch as it is used as a representation of the latter.^22^ The possibility to represent intensity of sensation in terms of the intensity of the stimulus is warranted by the mapping expressed by the measurement formula, which describes the relationship between these two quantities with respect to a concrete measurement system, that is, the human body. The choice of intensity of stimulus as the other term of the functional-representational relationship is indeed a conventional one, but the choice of a convention is only a part of the story. Indeed, Fechner’s measurement formula established a correlation between the intensity of stimulus taken as a representing quantity, and the intensity of sensation as the represented quantity. Yet, in Fechner’s view, the importance of the measurement formula went beyond a mere correlational aspect. From the perspective of his inner psychophysics, as we have seen, the measurement law was itself justified by the causal relationship between psychophysical excitation and intensity of sensation, a relationship that was yet to be empirically discovered.

The innovative character of Fechner’s correlative view of measurement had an influence that went well beyond the field of psychophysics. Notably, Fechner’s correlative view was taken by the physicist Ernst Mach as a blueprint for his own view of measurement (Heidelberger, 1993, 2004, 2010; Briggs, 2021; Staley, 2021). In Mach’s (1896/1986) view, measuring does not amount to discovering a state of the matter, but rather to discovering the relation holding between the measured characteristic and a chosen measurement standard.^23^ Particularly in the early stages of developing measurement procedures, the choice of measurement instruments and standards is conventional and guided by pragmatic considerations. Yet, by putting some sort of measurement standard in place, it enables the collection of empirical data that then allow for further empirical investigation of the relationship among the quantities that was somewhat postulated in the first place. This relational and iterative understanding influenced, in more recent times, the coherentist perspectives on measurement progress that I introduced in section “2 Epistemic circularity and psychological measurement,” especially Chang’s (2004) view of progress through epistemic iteration.

Before turning to my assessment of Fechner’s approach vis-à-vis subsequent developments in psychophysics, I must address two further points. The first is that the transition toward quantitative science that was characterizing German and, more generally, European science at the time constituted a central influence on Fechner’s approach to measurement. Most importantly, Fechner was working within the so-called *Euclidean* tradition of measuring magnitudes, according to which “ratios of magnitudes are equal to ratios of natural numbers or are approximated by ratios of natural numbers” (Zudini, 2011: p. 76).^24^ In other words, Fechner’s underlying conception of measurement was shaped by this classical understanding of measurement, by which all measurement requires quantification on a ratio scale, thus necessitating an absolute zero point and equality of intervals among units of the measurement scale.^25^ Therefore, the Euclidean model constrained the range of possible measurement scales that Fechner could choose to develop his measurement scale, and inevitably led him to strive for a quantitative approach that would enable him to measure intensity of sensation on a ratio scale.

Second, it is crucial to emphasize that Fechner’s epistemic goal, much as it was shaped by the search for precise quantification, was not that of discovering the *ultimate* quantitative model of human sensory experience. In fact, Fechner used mathematical tools not only with the aim of representing quantitative relationships, but also as investigative tools, for example in the case of his statistical notion of threshold (Marshall, 1982). In this respect, mathematization was certainly a goal for Fechner, but not in the sense of providing a quantitative description of human experience that would not require further refinement. This is demonstrated by the fact that Fechner was very much aware of the provisional character of his quantification, since he regarded his *Elemente* more as a research progress report than as a final scientific product (Fechner, 1860, vii). In addition, in his treatise he recognizes the absence of practical alternatives to taking the intensity of the stimulus as a concrete standard to quantify sensation, and he emphasizes that the main role of theorizing is its function of generating testable assumptions, rather than of providing incontrovertible definitions. All these points suggest that his goal of achieving mathematical tractability for the supposed quantitative phenomenon under investigation was very much open to the possibility of refining his formal characterization through empirical considerations made available by further investigations. Indeed, to establish a standard for measuring sensation intensity Fechner had to resort to a number of conventional choices, most notably that of the equality of jnds. Yet, it was very clear to him that these specific choices were only pragmatically necessary and that they were revisable in the light of empirical evidence.

In short, the ideal of universal quantification spreading fast in the nineteenth century science pushed Fechner toward the goal of providing an overarching model of quantification of sensation. This required embracing core untested assumptions, such as the one concerning the quantifiability of sensation, that were modeled on physical quantification. Fechner developed an original approach to devise a measurement standard based on his correlational view of measurement. This approach required him to make untested assumptions about the quantitative structure of the characteristic of interest, in order to overcome the dead-end of circularity. The non-testability of the causal complement to his correlative measurement formula and the issues raised by the quantity objection are indeed crucial unresolved aspects of his approach to measurement. Yet, several features of Fechner’s epistemic attitude, such as his recognition of the absence of practical alternatives to his chosen standard and of the revisability of his standards based on empirical considerations, show the modernity of his epistemological standpoint. Viewed from this perspective, Fechner’s own approach to measurement seems to resonate with more recent approaches to construct validity in psychological measurement and coherentist views in epistemology of measurement. This is because these approaches emphasize that progress along any of the interacting dimensions of theory, experimentation, and measurement should reverberate on the network of assumptions and empirical generalizations involved in the definition of quantities and units.

### 4.4 Fechner’s standard as a first epistemic iteration for psychophysical measurement

In the previous paragraphs, we have seen that the empirical validity of Fechner’s quantification of sensation has been rescaled in the light of twentieth century developments in psychophysics. Most importantly, while his assumption of the equality of the jnds was empirically disproved, his logarithmic measurement law was found to hold only for a restricted range of sensory modalities. Therefore, from our vantage point, Fechner’s overall project of quantifying sensation might be regarded as an unsuccessful enterprise. Yet, if we take a view from “above,” a different assessment is possible.

First of all, Fechner’s methods of experimentation and statistical analysis, through which he located the jnds and assessed the sensitivity of human discrimination, were universally adopted (Stigler, 1986; Briggs, 2021). In addition, several commentators have emphasized that Fechner’s construction of a measurement standard for intensity of sensation actually enabled the subsequent advancement of psychophysical measurement (e.g., Falmagne, 2002; Heidelberger, 2004; Isaac, 2013). Fechner’s way out of the circularity issue made it possible to treat psychophysical data mathematically, thus enabling scientists to gather more empirical knowledge and develop more advanced measurement techniques, such as multidimensional scaling (cf. Isaac, 2013, 2017). This, in turn, enhanced the empirical investigation of the quantitative relationships among jnds and made it possible to replace Fechner’s standards in light of empirical considerations. More precisely, the fact that Fechner put a measurement standard in place opened the door for the mathematical analysis of psychophysical data. This enabled the generation of precise predictions about just noticeable differences, which could then be empirically tested, thus enabling the refinement of the measurement standard itself at a later stage.

In sum, Fechner’s engagement with the issue of measurement circularity led him to a quantification of sensation that achieved sufficient mathematical tractability to start off a long-term process of refinement of the measurement standards for intensity of sensation over time. The measurement outcomes obtained through Fechner’s quantification were, in fact, taken as the empirical basis of a process that, in the *longue durée*, enabled the study of the quantitative relationships among jnds and led to the development of more accurate standards, thus making psychophysics the empirically successful research program that is today. From this point of view, Fechner’s quantification can be considered as successful insofar as it satisfied the goal of providing a first measurement standard for sensation intensity, even if its empirical adequacy was later found to be limited. In this respect, Fechner’s standard represents a first epistemic iteration in the process of developing psychophysical measurement. His approach to the problem of measurement circularity, with its strengths and limitations, served the purpose of overcoming an impasse and providing a first, temporary standard which could then be refined over time.

## 5 The relevance of Fechner to current methodology of psychological measurement

So far, I have analyzed the approach to the epistemic circularity behind Fechner’s quantification of sensation. I have discussed how Fechner stabilized the circularity by making a number of assumptions, which concerned the subject matter that he was attempting to quantitatively model, its relationship with a spatio-temporally located standard, and the notion of measurement itself. I contextualized the development of Fechner’s quantification from within the framework of nineteenth-century science, and I emphasized that the consolidating ideals of quantitative objectivity and universality were built into his creation of a measurement standard for sensation intensity. Nevertheless, I stressed that his approach to measurement, and to the circularity issue specifically, had innovative aspects, which resulted from Fechner’s appreciation of the subjective aspect of measurement, both methodologically and epistemologically.

Finally, I have shown Fechner’s contribution can fit a story of success, to the extent that we regard his approach to circular measurement as conducive to a first, albeit imperfect, standard for measuring sensation, which could start a process of epistemic iteration. Taking this perspective seems also justified by the relationship of Fechner’s own conceptualization of measurement with coeval perspectives that were embracing some form of coherentism about measurement. In addition to Fechner’s influence on Mach, Briggs (2021) emphasizes that Fechner was working at a time in which Maxwell and Thomson (also known as Lord Kelvin) were actively reflecting on how advancements in physical measurement are carried forward by the identification of the proper measurement laws. In this sense, Maxwell and Thomson were envisioning a coherent system of fundamental and derived units defined by referring to a set of constants of nature, thus preconizing the approach currently taken by the International System of Units (cf. de Courtenay, 2022).

In the context of psychology, it has been argued that this “Maxwellian” approach has been insufficiently considered by methodologists of measurement, to the benefit of traditions such as operationalism and representationalism (McGrane, 2015). Fechner was himself a forerunner of this approach, in that he developed his measurement standard by identifying a measurement formula that functionally related internal sensation to a spatio-temporal property, i.e., the intensity of the physical stimulus. Indeed, his formula was only correlational, since the functional relationship was not based on an empirical causal law, but only on a statistically modeled set of observations used to infer the magnitude of the characteristic of interest. As we have seen, however, the causal law was, in his view, to be eventually identified empirically by research in inner psychophysics, which would provide the final validation to the relationship underlying his measurement standard. While this validation has not been provided, this aspect of Fechner’s approach seems to be the carrier of an optimistic vision of measurement, “one that reflects the ongoing efforts to uncover and understand the causal mechanism underlying the relationship” (Briggs, 2021: p. 52).

Even if we grant that Fechner’s vision may hold for psychophysics, the approach based on identifying an empirical causal law that justifies the representational relationship in measurement might not be regarded with the same optimism in most of quantitative psychology. This is because, “[c]ontrary to beliefs widespread in psychology, findings about individuals’ perceptions of physical phenomena cannot be generalized to all psychical phenomena, which, given their non-spatial properties, differ fundamentally from the spatially extended phenomena the perception of which is studied in psychophysics” (Uher, 2019: p. 242). In other words, the fact that there are no evident observable properties that can be linked to the psychological characteristics that we aim to measure may be considered as an intrinsic barrier to this approach. This is because most psychological instruments are not based on the detection of some perceptible quality, as in the case of sensations of physical stimuli. Instead, they are necessarily based on language, thus involving interactions between the human instrument (i.e., the rater), the non-human instrument, and the phenomena and properties under investigation. Interpretive decisions, rather than empirical causal relationship, are therefore required to establish a representational relationship in these measurement systems (Uher, 2021a).

The question then is the following: To what extent, if at all, can we apply insights from Fechner’s psychophysical measurement to current quantitative psychology? On the one hand, his approach to the development of a measurement standard has been praised for its radically innovative epistemological import. On the other, it has been regarded as intrinsically flawed or unsuitable to most needs of quantitative psychology. In my view, the relevance of Fechner for current issues in quantitative psychology should be searched neither in his specific way of developing a measurement standard for sensation, nor in his theory of measurement *per se*. As such, we cannot take the success story of psychophysics, and of Fechner’s role in it, as grounds for optimism with respect to the possibility of achieving a similar form of quantification in the rest of psychology. Yet, the strengths and limitations of his general epistemic attitude toward measurement can provide important reflections for current quantitative psychology. Most importantly, his approach toward the problem of measurement circularity gives us the possibility to rethink important epistemic categories central to the assessment of measurement in current psychology, such as the notion of successful measurement and the notions of validity and objectivity.

A first point concerns the goal of stabilizing the circularity. As I have shown, in Fechner’s approach, the assumption of a shared human physiology functioned as an essential anchor to achieve stabilization. The presence of an idealizing element opens up a question concerning both the justification for this idealization and its implications. Clearly, this assumption was not taken for granted in other contexts of psychophysical research at later stages, whereby *differences* among sensory experiences of individuals, rather than their similarities, became relevant. For instance, this occurred when it started becoming clear that “individual variations in sense experience approached but did not quite align with the new biological theories of human variation powered by the concept of heredity” (Fretwell, 2020: p. 3). In this sense, while idealizing assumptions such as this one might be necessary to stabilize the circularity, it is crucial to clearly identify the scope of their justification. This is very relevant to psychological measurement in general, inasmuch as a certain measurement tool is aimed, for instance, at tracing differences within populations (e.g., distinguishing among human groups according to personality traits). When the goal is that of identifying differences, rather than broad generalizations, it becomes very difficult to find justification for such strong idealizations.

Most importantly, Fechner’s need to stabilize the circularity derived from his epistemic goal of identifying a first measurement standard for sensation intensity. This, in turn, involved a trade-off of epistemic values, which is relevant to the assessment of what counts as successful measurement. The adoption of idealizing assumptions about the measurand, the measurement instrument, and their relationship, is always required to model the measurement process (Tal, 2017). These idealizations serve the purposes of model tractability, but this occurs to the detriment of the possibility of achieving complete representational accuracy, which is itself an idealization (Teller, 2013, 2018). By assuming a shared human physiology, Fechner could dispense with accounting for individual neurophysiological variability of test subjects and could experiment mostly on himself, thus privileging generality of representational accuracy.

The insight that can be taken from Fechner’s use of untested or untestable idealizations is that these assumptions can be necessary to stabilize the circularity and enable the development of a new measurement standard. As such, these assumptions can be crucial to successful measurement, where success should be understood as relative to the purpose at hand and to the trade-off of epistemic values that it underlies. In the case of Fechner, the use of this idealization was conducive to achieving mathematical tractability of psychophysical phenomena. This achievement was not itself sufficient with respect to the overarching goal of providing a universal, empirically adequate quantitative representation of intensity of sensation, but it did enable the improvement of the measurement standard at a later stage of psychophysics. Yet, Fechner did not explicitly acknowledge the use of this idealized assumption with reference to the achievement of a specific goal, nor did he clearly acknowledge the validity of the resulting measurements as context-dependent.

This insight can be relevant to current quantitative psychology quite independently from the stance concerning where research efforts should be directed to improve the standards of psychological measurement. Indeed, many voices have pleaded for more and better theorizing in psychology in the wake of the replication crisis (e.g., Muthukrishna and Henrich, 2019; Oberauer and Lewandowsky, 2019). However, how should this plea be tailored to address measurement circularity in psychometrics? Among the challenges of psychometrics, we find, for example, the fact that standardized questionnaire statements are interpreted very differently across test subject, and even by the same subject in different circumstances (e.g., Lundmann and Villadsen, 2016). If we take the empirical identification of a causal quantitative relationships underlying the existing measurement standards as our goal (e.g., Kellen et al., 2021), then we should strive for a better theoretical and causal understanding of the measurement instruments used, in particular the language-based reports with which psychology cannot dispense (Uher, 2021b). Indeed, the multi-dimensionality and instability of the psychological subject matter, as well as the availability of intraquestive methods only, call for searching something quite different from the single causal law that Fechner thought could justify his measurement standard. For example, an important contribution in this direction would be to better identify which conditions affect the interpretations given by test subjects (i.e., the humans as measurement instruments) to the items of standardized assessment tools, and how this feeds back into converting resulting information into fixed scales.^26^ While such an effort is made, however, current standards from which such research is conducted would still presuppose idealized untested or untestable assumptions about the causal quantitative relationships. The story of psychophysics and Fechner tells us that these assumptions can play an important role in the long run, but that their scope of application and impact on the validity of measurement must be carefully assessed, especially by making explicit the measurement goals and the related value trade-offs.

The second, related point concerns the categories of validity and objectivity that result from such a picture. Indeed, coherentist epistemologists of measurement have suggested that “quantitative structure is ultimately established through a coherentist fit between substantive theory and data that leads to improvements in various desiderata such as the scope, accuracy, and fruitfulness of the relevant inquiry. The process of establishing such coherence involves bottom-up discovery of relations in data alongside top-down, theory-driven corrections to the data” (Tal, 2021: p. 735). In other words, the process of refinement of measurement standards over time involves the progressive establishment of quantitative structure through coordinated improvements at the level of theory and of data which, in turn, can be evaluated as improvements thanks to reference to certain values. As I mentioned, the identification of quantitative structure would occur differently in psychometrics compared not only to the natural sciences, but also to psychophysics. In this sense, coherentism can be a helpful epistemological approach for quantitative psychology, if the search of a coherentist fit is not merely mimicked from paradigmatic cases in the physical sciences, but it is adequately paraphrased to the context of psychological measurement.

Most importantly, a proper characterization and focus on measurement circularity should be central to efforts in this direction. To understand exactly how, the story of Fechner’s measurement standard for subsequent psychophysics reminds us of two important points. First, that quantification is open-ended, since it will always be possible to perfect the knowledge of quantities and of the relationships among them as science further progresses (cf. Riordan, 2015). Second, that the epistemic goals of quantification change over time, in parallel with changes and improvements in measurement standards and techniques, thus changing, in turn, the criteria for evaluating what counts as a successful, adequate, or useful form of measurement or quantification.^27^ Therefore, rather than considering the circularity as an issue to be solved once and for all by means of an ultimate standard, methodologists should “listen” to what methodological and epistemological questions emerge in connection with the appearance of a specific form circularity in a specific context of inquiry. This, in turn, would open up questions concerning the values (epistemic or not) that are embedded in a certain measurement practice and the related trade-offs which, as we have seen, are related to the goals that are pursued by trying to achieve a certain, temporary solution to the measurement circularity.

In this sense, focusing on measurement circularity can be conceived as a hermeneutic tool (McClimans, 2023), which does not serve the only purpose of identifying rigid causal relationships that justify the quantitative structure of a measurable characteristic, at least in the short run. Rather, this tool is useful to reflect on the conditions of scope, accuracy and fruitfulness of a certain measurement standard, in other words, on the criteria of success that a scientific community wants to pursue by finding a certain, temporary solution to the circularity. In this sense, acknowledging the trade-offs of values can also mitigate some limitations of coherentist approaches when applied to subjective evaluations and the human sciences (cf. Thompson, 2023). By explicitly identifying what a certain solution to measurement circularity does and does not fulfill, the purposivity and selectivity of a measurement standard is acknowledged and the relative validity of the resulting measurements fully recognized. Such an understanding of validity as context-relative and purpose-oriented is very much in line with current standards (American Educational Research Association et al., 2014), and indeed it calls for a notion of objectivity that, when applied to measurement, will look quite different from the one that was guiding Fechner. By putting the subject back in the measurement process, Fechner initiated a process that, despite his convictions, requires us to acknowledge and integrate goals and values to objectively evaluate our measurement of the human.

## Author contributions

ML: Writing – original draft, Writing – review & editing.

## Footnotes

1 The distinction between direct and indirect measurement methods is neither univocal nor uncontroversial. According to certain measurement traditions, this distinction collapses even in the case of intuitively direct physical measurements, e.g.: “ […] all measurements are indirect in one sense or another. Not even simple physical measurements are direct, as the philosophically naïve individual is likely to maintain. The physical weight of an object is customarily determined by watching a pointer on a scale. No one could truthfully say that he ‘saw’ the weight.” (Guilford, 1936: p. 3).

2 Heidelberger (2004) offers a comprehensive account of Fechner’s philosophy, including his philosophy of science and his theory of measurement. Briggs (2021) focuses more specifically on Fechner’s meta-perspective on measurement and several technical aspects of great epistemological relevance.

3 For instance, conceptual errors involved by naïve uses of verbal items as measurement scales raise concerns that are distinctive to psychological measurement. Cf., for instance, Lundmann and Villadsen (2016), Smedslund (2016), and Uher (2022).

4 Another example of difference in challenges between psychophysics and other areas of psychology comes from the phenomenon of reactivity, i.e., the fact that humans may respond to their awareness of being studied, which manifests itself differently in different psychological contexts of research (e.g., Orne, 1962; Feest, 2022b). As such, reactivity is plausibly lower in the context of measuring sensory reactions than when the measurement process involves more complex language-based abilities, as in the case of higher-order psychological properties. On the pervasiveness of reactivity in the human sciences see Marchionni et al. (2024) and references therein.

5 See, for instance, Uher (2021b) for a comprehensive analysis of the conceptual and epistemological challenges to contemporary psychological measurement.

6 Chang (2004) labels this issue the “problem of nomic measurement” (cf. also Sherry, 2011; Bradburn et al., 2017), while van Fraassen calls it the “problem of coordination,” following an epistemological tradition that dates back to the turn of the twentieth century (Mach, 1896/1986; Reichenbach, 1920. Cf. Padovani, 2017 for a discussion).

7 The picture provides a general description of an epistemic problem. This description abstracts away from the specific measurement system (i.e., the concrete measurement procedure and the theoretical model of the measurement process), as well as from the measurement target under investigation. For examples of epistemological analyses of this problem in different scientific disciplines, see the references in footnote 8.

8 These include, among others, medical measurement (McClimans, 2013), physical anthropology (Luchetti, 2022), perception studies (Barwich and Chang, 2015), and psychometrics (McClimans et al., 2017).

9 The reason for this difficulty is that the historical development of successful measurement procedures for a certain quantity or property is often intertwined with the empirical process of identification, confirmation and refinement of the relevant measurement laws that are required to infer information on the measurand from the result of a measurement process (Chang, 2004; Riordan, 2015; Luchetti, 2020). Yet, during calibration, i.e., the modeling of a measurement process, these empirical regularities are usually taken as fixed background presuppositions that justify the measurement inference. Therefore, the calibration and standardization of measurement procedures are often performed with only a partial knowledge of the necessary theoretical background (Barwich and Chang, 2015; Tal, 2017).

10 Validity as a technical term in this sense was first explicitly introduced in the context of attempts at standardizing intelligence testing in the 1920s, but it was progressively adopted as a methodological notion in domains beyond psychology and education. Even though validity in its original sense is commonly agreed to indicate the extent to which the assessment of an item is informative about the characteristic of interest, these developments led to a proliferation of validity concepts and taxonomies (cf. Newton and Shaw, 2014; Slaney, 2017). See, for example, Borsboom et al. (2004) and Markus and Borsboom (2013) for an overview of contemporary debates surrounding validity in psychometrics.

11 As of today, the unitary understanding of validity adopted, for instance, by US Standards in psychology and education is inspired by the construct validity perspective, even though it includes evidence from sources that were previously related to other validity notions (cf. American Educational Research Association et al., 2014).

12 See, for instance, Borsboom et al. (2009) for a criticism of construct validity from within psychometrics; Slaney and Garcia (2015) for a discussion of the use of “construct” language in psychology; Alexandrova and Haybron (2016) for a philosophical critique of the notion of construct validity; Stone (2019), Feest (2020), and Zhao (2023) for recent philosophical perspectives.

13 These perspectives aim to account for the fact that “screening and testing of participants as measuring instruments are absolutely necessary for reliable and valid psychological measurement” (Berglund et al., 2013), thus emphasizing an underappreciated dimension of analysis in the epistemology of psychological measurement. While the psychometric approach to measurement has developed fruitful tools to address this dimension, such as the Rasch model, this has been unevenly recognized within both psychology and the epistemology of measurement (e.g., McClimans et al., 2017).

14 The concept of threshold in psychology was introduced by Herbart (1824–1825), who defined it mathematically. For a historical overview of the notion of threshold, see Corso (1963).

15 Fechner used this empirical result to construct a unit for a measurement scale of sensation intensity already in 1851, without referencing Weber. Only later he referred to this regularity as “Weber’s law.”

16 This method became later known as the method of constant stimulus (cf. Brown and Thomson, 1921; Guilford, 1936).

17 For a discussion of the epistemological implications of this modeling assumption, see Briggs (2021: p. 39–41).

18 For a detailed account of the criticisms against Fechner’s quantification of sensation from his contemporaries, see Heidelberger (2004: p. 207–234). Cohen’s neo-Kantian objection to Fechner’s quantification and its impact on subsequent neo-Kantian philosophy are discussed by Giovanelli (2017). Feest (2021) reviews the objections raised by Gestalt psychologists against Fechner’s additivity assumption. Biagioli (2023) discusses the relationship between Fechner’s quantification and Helmholtz’s view of sensory measurement.

19 See Briggs (2021: p. 51–55) for an excellent discussion of the quantity objection and its implications.

20 The first identification of a power relationship for the dependence of visual acuity on the intensity of the light by which the stimulus pattern was illuminated dates back to the work of Tobias Mayer in 1754 (Grüsser, 1993).

21 This is a move that he had already made when conducting his inquiry on Ohm’s law and the Galvanic circuit (Fechner, 1831; cf. also Marshall, 1990).

22 The conventionality of this move leads to define new units for the physical stimulus, a result that was criticized by Boring (1921).

23 When Mach (1872/1909) urged that the proper aim of science is to discover the fixed functional dependence of phenomena on one another, he was following the lead of Fechner (cf. Ryckman, 1991).

24 For brief characterizations of the Euclidean tradition and its historical significance see, for instance, Mari (2013).

25 This view of measurement was to be abandoned by subsequent approaches to psychological measurement while a strict identification between measurement and quantification has largely been discarded as of today.

26 A recent example in this sense can be found in recent research on neurodegeneration, which has formulated metrological references for cognitive task difficulty that can be used to calibrate the measurement system function (Melin et al., 2021).

27 For an example of a recent perspective that emphasizes the relevance of goal-orientedness of quantification, and measurement in general, see Irribarra (2021, especially Ch. 4).
